# Physiology and Emerging Biochemistry of the Glucagon-Like Peptide-1 Receptor

**DOI:** 10.1155/2012/470851

**Published:** 2012-05-14

**Authors:** Francis S. Willard, Kyle W. Sloop

**Affiliations:** ^1^Translational Science and Technologies, Lilly Research Laboratories, Eli Lilly and Company, Indianapolis, IN 46285, USA; ^2^Endocrine Discovery, Lilly Research Laboratories, Eli Lilly and Company, Indianapolis, IN 46285, USA

## Abstract

The glucagon-like peptide-1 (GLP-1) receptor is one of the best validated therapeutic targets for the treatment of type 2 diabetes mellitus (T2DM). Over several years, the accumulation of basic, translational, and clinical research helped define the physiologic roles of GLP-1 and its receptor in regulating glucose homeostasis and energy metabolism. These efforts provided much of the foundation for pharmaceutical development of the GLP-1 receptor peptide agonists, exenatide and liraglutide, as novel medicines for patients suffering from T2DM. Now, much attention is focused on better understanding the molecular mechanisms involved in ligand induced signaling of the GLP-1 receptor. For example, advancements in biophysical and structural biology techniques are being applied in attempts to more precisely determine ligand binding and receptor occupancy characteristics at the atomic level. These efforts should better inform three-dimensional modeling of the GLP-1 receptor that will help inspire more rational approaches to identify and optimize small molecule agonists or allosteric modulators targeting the GLP-1 receptor. This article reviews GLP-1 receptor physiology with an emphasis on GLP-1 induced signaling mechanisms in order to highlight new molecular strategies that help determine desired pharmacologic characteristics for guiding development of future nonpeptide GLP-1 receptor activators.

## 1. Introduction

The glucoregulatory role of the gut is demonstrated by studies showing insulin secretion is profoundly more robust following glucose ingestion compared to the insulinotropic response achieved by parenteral administration of intravenously infused glucose [1–5]. This physiologic phenomenon, coined the “incretin effect,” is primarily mediated by two enteric factors known as the incretins: glucagon-like peptide-1 (7-37)/(7-36)-amide (GLP-1) and glucose dependent insulinotropic polypeptide (1-42) (GIP) [6–13]. In addition to glucose, the presence of other macronutrients in a mixed meal, such as lipids and amino acids, in the intestinal lumen stimulate a similar incretin response [14, 15]. When moving through the intestine, nutrients interact directly with sensory receptors and integral membrane channel and transporter proteins localized on the microvillus-rich apical membrane surface of open-type endocrine cells. These cells are embedded in the mucosal lining throughout various regions of the intestinal tract and release the incretins upon nutrient stimulation. In L-cells, located throughout the intestine but predominantly found in the ileum of the distal small intestine and colon [16, 17], GLP-1 is produced by posttranslational cleavage of the 160-amino acid proglucaon precursor protein [18, 19], a process requiring prohormone convertase-1/3 [20–22]. GIP is the single peptide derived from proteolytic processing of a 153-amino acid precursor protein [23] expressed in endocrine K-cells located mainly in the duodenum and proximal jejunum of the upper small intestine [24].

### 1.1. Physiologic Action of GLP-1

Upon release into the circulation, GLP-1 and GIP facilitate glucose disposal by directly acting on pancreatic islets to enhance postprandial insulin secretion [6–13]. This process is mediated by two class B1 (secretin-like family) seven transmembrane spanning, heterotrimeric G-protein coupled receptors (GPCRs) that signal in response to binding and occupancy by GLP-1 [25, 26] and GIP [27], respectively. Both of these GPCRs predominantly couple to the G*α*_s_ subunit which activates adenylyl cyclases to increase intracellular cyclic 3′5′AMP (cAMP). Genetic deletion of both receptors in mice leads to glucose intolerance and defects in glucose stimulated insulin secretion [28]. In addition to ligand stimulated cAMP generation, *β*-arrestin interaction [29, 30] and signaling pathways that mobilize intracellular calcium are important effectors of incretin action [31, 32].

In humans, the incretin effect is often reduced in patients suffering from type 2 diabetes mellitus (T2DM) [33]. To combat this, initial strategies to develop incretin based “replacement” therapies largely focused on GLP-1 receptor analogs because studies suggested diabetic patients are resistant to GIP treatment [34, 35], while GLP-1 infusion elicits a strong insulin secretory response and can normalize hyperglycemia [36–39]. In contrast to GIP, GLP-1 also induces several additional antidiabetic effects, including inhibition of glucagon secretion [6, 8] and gastric emptying [40–42] (which both help improve postprandial glucose control) and a decrease in appetite and food intake [43–47]. These latter effects are mediated by the GLP-1 receptor expressed in extrapancreatic tissues, most notably those of the gastrointestinal tract and central nervous system.

While infusion regimens demonstrate remarkable antidiabetic pharmacology, elimination metabolism and pharmacokinetic characteristics of native GLP-1 present major hurdles to developing it as an effective pharmaceutical agent. One significant challenge in pursing GLP-1 based molecules is that GLP-1 is rapidly inactivated by dipeptidyl peptidase 4 (DPP4), a plasma membrane bound enzyme that is positioned with its active site orientated towards the extracellular space. This ubiquitously expressed “ectopeptidase” cleaves the N-terminal dipeptide, His^7^-Ala^8^, to inactivate GLP-1 [48, 49]. Removal of these residues dramatically reduces the binding affinity of the peptide for the GLP-1 receptor, thus abolishing its ability to effectively activate receptor signaling [50]. DPP4 is highly expressed on the surface of endothelial cells lining blood vessels; consequently, GLP-1 is immediately vulnerable to inactivation following release into the circulation [51]. Upon cleavage, the inactive GLP-1 metabolite is eliminated by the kidney [52]. As a result of rapid postsecretory proteolysis and renal elimination, the biological half-life of GLP-1 is estimated to be between 1 to 2 minutes [53, 54]. These characteristics limit the pharmaceutical potential of native GLP-1.

### 1.2. GLP-1 Receptor Peptide Agonists

Several efforts pursued novel GLP-1 analogs with improved metabolic properties. A common approach was to introduce N-terminally substituted modifications to reduce DPP4 sensitivity [54–56]. To date, attempts solely focused on amino acid substitutions of native GLP-1 to identify longer acting molecules likely have been hampered by other issues such as renal clearance and secondary degradation by other endopeptidases. However, two alternate approaches proved successful in advancing more stable, degradation resistant GLP-1 receptor agonists. Both exenatide and liraglutide are approved for marketing by several government regulatory agencies for the treatment of T2DM.

Exenatide is a 39-amino acid peptide GLP-1 receptor agonist that is fully efficacious in cellular assays and competitive with native GLP-1 in receptor binding studies [26, 57–59]. It is the synthetic version of exendin-4 which was among several bioactive peptides containing an N-terminal histidine identified in crude venom preparations extracted from perimandibular salivary glands of Helodermatidae lizards [60, 61]. Exendin-4 was isolated from the poisonous venom of the Gila monster, *Heloderma suspectum* [60], a lizard indigenous to the southwest United States in the Gila River area of New Mexico and Arizona [62]. In addition to mimicking the physiologic glucoregulatory actions of native GLP-1, exendin-4 is a poor DPP4 substrate [63] and is cleared from the body primarily by glomerular filtration in the kidney [64, 65]. Consequently, exenatide has a longer duration of action compared to GLP-1 [66–68] and an estimated biological half-life of approximately 4 hours [64, 69]. In April of 2005, under the brand name *Byetta*, exenatide became the first enteroendocrine based therapeutic approved by the United States Food and Drug Administration (FDA) for the treatment of T2DM.

The second GLP-1 receptor agonist approved to treat T2DM is liraglutide (NN2211). For this molecule, a “fatty acid derivatization” strategy was used to prolong the *in vivo* action of GLP-1. This approach attaches a fatty acid moiety to GLP-1 in order to facilitate GLP-1 binding to serum albumin. Liraglutide is acylated on Lys^26^ with a covalently attached palmitoyl (C16:0) chain [70]. As this modification enables binding to albumin, GLP-1 is then sterically protected from DPP4 degradation [70]. The plasma half-life of liraglutide is estimated to be between 11 and 15 hours [71, 72]. Under the brand name *Victoza*, liraglutide received marketing approval by the FDA in January of 2010 for the treatment of T2DM. *Byetta *and *Victoza *are both commonly prescribed medicines.

## 2. GLP-1 Receptor Signal Transduction and Second Messenger Pathways

As the best characterized *in vivo *action of GLP-1 is an acute insulinotropic effect mediated by the GLP-1 receptor in pancreatic *β*-cells, the signal transduction coupling mechanisms of this receptor primarily have been analyzed using *ex vivo* preparations of pancreatic islets, transformed pancreatic *β*-cell lines, and recombinant GLP-1 receptor expressing systems, Accordingly, critical evaluation of GLP-1 receptor signal transduction in extrapancreatic tissues can be made by inference only. Use of the various peptide GLP-1 receptor agonists to define the *in vitro* pharmacologic properties of the receptor should define key assay systems to enable optimizing small molecule GLP-1 receptor activators.

### 2.1. GLP-1 Receptor Activation

**Figure 1:**
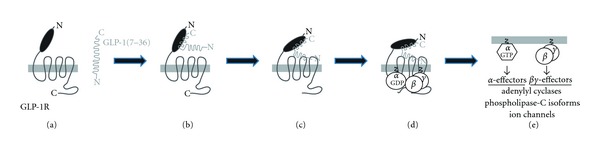
The activation mechanism of the GLP-1 receptor. Biochemical and structural studies have led to a model of class B1 GPCR activation by peptide hormones referred to as the “two-step” mechanism [82]. (a) In the unliganded state, the GLP-1 receptor (GLP-1R) is in a predominately inactive conformation. The natural ligand GLP-1(7-36)-NH_2_ is freely diffusible in solution and likely has substantial intrinsic *α*-helical structure. (b) An initial binding event between the globular ectodomain at the N-terminal of the GLP-1R and the C-terminal of the GLP-1(7-36)-NH_2_ peptide occurs. This “low affinity” interaction acts as a tether or “affinity trap” to localize GLP-1 at the GLP-1R. (c) The weak affinity of the N-terminus of GLP-1(7-36)-NH_2_ is then able to productively engage with transmembrane domain and loop residues of the receptor to induce a high affinity interaction and likely a conformational change in the GLP-1R. (d) Coincident with agonist binding, the G-protein bound conformation of the GLP-1R is stabilized. This represents the classic high affinity agonist bound state. (e) The high affinity agonist bound state is transient in an intact system as the GLP-1R stimulates guanine nucleotide exchange on the *α*-subunit of the G-protein heterotrimer, leading to G-protein dissociation and independent or synergistic activation of effector proteins by liberated G*α*·GTP and G*βγ*.

**Figure 2:**
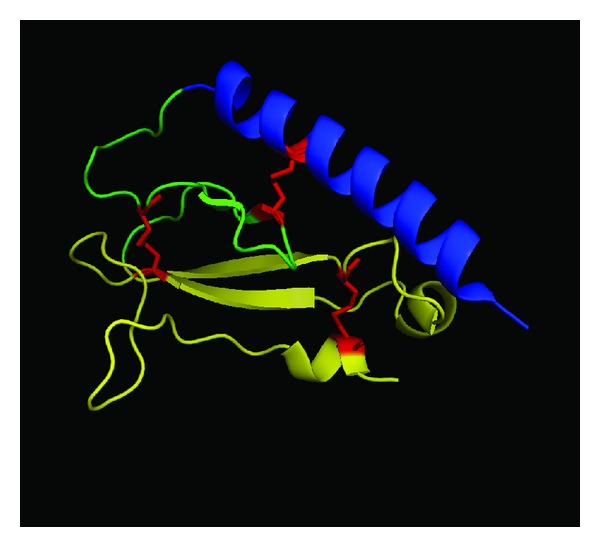
Structure of the GLP-1 receptor ectodomain. The overall structure of the GLP-1 receptor ectodomain is depicted (PDB ID: 3IOL) [74]. The tripartite *α*-*β*-*βα* structure is annotated using color; from N-terminal to C-terminal *α* (blue), *β* (green), *βα* (yellow). The three conserved disulfide bonds that stabilize the tertiary structure of the ectodomain are colored in red.

**Figure 3:**
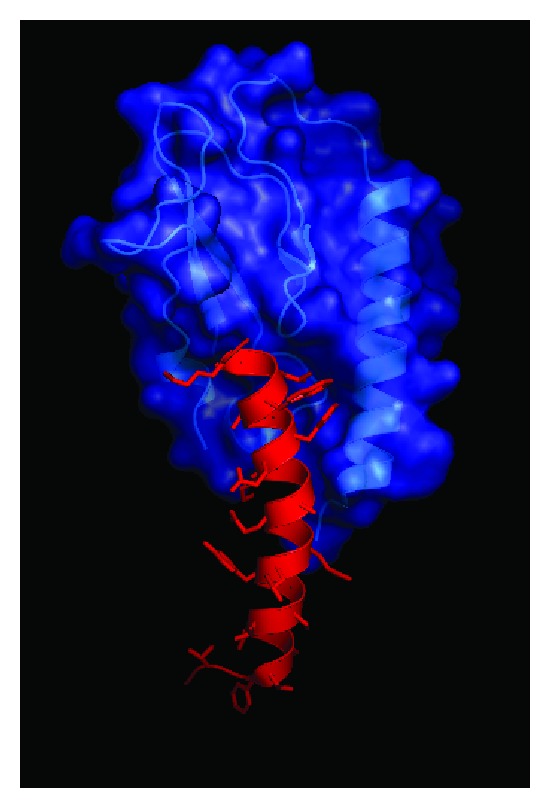
Structural determinants of ligand binding to the GLP-1 receptor ectodomain. (a) Structure of the GLP-1 receptor ectodomain bound to GLP-1. Blue ribbon and space filling model is GLP-1 receptor and the red ribbon and stick model is GLP-1. Data are derived from PDB ID: 3OL.

Both GLP-1 and exendin-4 are *α*-helical peptides that interact with the GLP-1 receptor by binding multiple extracellular contact points to induce receptor signaling [73–75]. Similar to the “two-step” mechanism proposed for other class B1 GPCRs [76] (Figure 1), the GLP-1 receptor utilizes an N-terminal extracellular domain as an “affinity trap” to recognize and bind peptide ligands [77, 78]. The N-terminal domain of the GLP-1 receptor is conserved among class B1 GPCRs forming an *α*-*β*-*βα* protein fold that has structural homology to the sushi/CCP/SCR protein folds [79, 80] (Figure 2). This structure, referred to as an “ectodomain” (ECD), is a trilayer fold composed of an N-terminal *α*-helix, a middle section of two antiparallel *β* strands, and a final lobe composed of two additional antiparallel *β* sheets and a short *α*-helical region (*βα*) (Figure 2). The overall structure of the ECD is stabilized by three pairs of disulfide bonds formed between six conserved cysteine residues that lock the three layers of the ECD together [81] (Figure 2). X-ray crystal structures of exendin-4 and GLP-1 bound to the ECD confirm the “affinity trap” hypothesis showing the C-terminal *α*-helical region of GLP-1 or exendin-4 is positioned within a binding cleft of the N-terminal ECD [74, 75] (Figure 3). Both GLP-1 and exendin-4 are amphipathic in nature, and this defines their structurally conserved interaction mechanism with the ECD. The hydrophobic faces of GLP-1 and exendin-4 make the majority of interactions with the ECD and likely are the key contributors to ECD/ligand affinity with only a minor contribution of binding energy provided by the hydrophilic regions of GLP-1 receptor agonist peptides [74, 75] (Figure 3).

The second step of the class B1 GPCR activation model predicts the ECD docks the bound peptide in a position that promotes direct contact of N-terminal residues of the ligand with the central activation pocket of the receptor, a region consisting of three interconnecting extracellular loops often referred to as the helical bundle or “J” (juxtamembrane) domain. GLP-1 (or exendin-4) binding to this core region induces a conformational rearrangement of the membrane-spanning *α*-helices, eliciting a shift of the intracellular receptor loops to stimulate intracellular signal transduction (Figure 1).

Structural information regarding GLP-1 receptor peptide ligands is available primarily from NMR studies. The data are consistent with other class B1 GPCR ligands [83] in that GLP-1(7-36) and exendin-4 peptides are likely *α*-helical in structure with disordered N-termini, although the artificial environment in which these studies are conducted must be used to qualify any interpretation of the experimental data [84, 85]. Structural studies of class B1 GPCR ECDs have been very informative regarding the molecular mechanisms of peptide ligand selectivity and have generated new hypotheses regarding class B1 GPCR activation mechanisms. However, the field awaits definitive structural data to explain how peptide agonists activate their cognate class B1 GPCRs.

### 2.2. G-Protein Coupling

The GLP-1 receptor primarily couples to the G*α*_s_ heterotrimeric G-protein. Upon ligand binding, the resulting conformational change activates intrinsic guanine nucleotide exchange factor activity of the receptor to catalyze release of bound GDP from the G*α*_s_. The G*α*_s_ then rapidly binds GTP which leads to dissociation of G*α*_s_ and G*βγ*, consequently activating downstream effector pathways. Activated G*α*_s_ allosterically stimulates membrane-associated adenylyl cyclases to catalyze conversion of ATP to cAMP, which acts as an intracellular second messenger mediating GLP-1 signaling.

Elevation of cAMP in the pancreatic *β*-cell is a critical event in the process of glucose dependent insulin secretion and is likely the key mechanism by which GLP-1 and exendin-4 act on *β*-cells to potentiate insulin secretion [25, 26, 86]. However, early reports highlighted the ability of the GLP-1 receptor to couple to alternative signaling pathways, including phospholipase C (PLC) and the mobilization of intracellular Ca^2+^ [87, 88], consistent with the known effects of GLP-1 to stimulate Ca^2+^ mobilization in *β*-cells [89]. Further, multiple reports indicate GLP-1 receptor couples to Ca^2+^ mobilization when heterologously expressed [90, 91]. In these systems, it is generally assumed Ca^2+^ mobilization is a G*α*_q_ mediated process. In support of this, studies utilizing the azidoanilide-GTP cross-linking method show the GLP-1 receptor can cause activation of the G*α*_q_- and G*α*_i_-families of G-proteins in GLP-1 receptor expressing CHO cells [88]. Conversely, recent experiments using membrane GTP*γ*S binding assays demonstrate GLP-1 receptor activation does not induce measurable activation of G*α*_q_ or G*α*_i_ despite the presence of substantial PLC independent Ca^2+^ mobilization in GLP-1 receptor expressing HEK cells [91]. *In vivo*, pancreatic *β*-cell specific dual inactivation of G*α*_q_ and G*α*_11_ does not affect GLP-1 potentiation of glucose stimulated insulin secretion [92], whereas insulinotropic action through known G*α*_q/11_ coupled GPCRs, GPR40 and the M_3_ muscarinic receptor, is ablated. While this study elegantly demonstrates that G*α*_q/11_ signal transduction is not required for GLP-1 receptor mediated insulin secretion (using perifused islets), it is problematic that a single dose of 100 *μ*M GLP-1 (a concentration greater than 5 orders of magnitude above the *K*_*d*_ and peak circulating levels of active GLP-1) was used in the studies [49, 92].

While a role for G*α*_q/11_ signal transduction in *β*-cell GLP-1 receptor action is generally excluded, a PLC and Ca^2+^ mobilization pathway may be operant. Experiments using mouse *β*-cells indicate the elevation of cAMP by GLP-1 receptor signaling results in activated EPAC2 that stimulates PLC [31] and Ca^2+^ channel recruitment [93] to facilitate calcium induced calcium release, a process integral for robust insulin secretion. These data provide a potential mechanism whereby sole activation of the G*α*_s_ pathway induces cAMP- and PLC/Ca^2+^ dependent responses in *β*-cells. In light of the contrasting data, it is apparent that the phenotype of GLP-1 receptor signaling may differ according to the cellular context in which the receptor is expressed, a phenomenon now widely recognized but not well understood within the GPCR field [94]. Accordingly, the definitive *in vivo* G-protein coupling profile of the GLP-1 receptor is unclear, although G*α*_s_ induced cAMP accumulation is certainly integral to the biological response of GLP-1 receptor activation. It is of interest that other class B1 GPCRs demonstrate physiologically relevant coupling to multiple G-proteins; the parathyroid hormone receptor is the best characterized example [95]. The use of genetically modified mice, RNA interference, or novel pharmacological tools such as the G*α*_q/11_ inhibitor YM-254890 [96] may serve to answer whether the GLP-1 receptor functionally couples to G-proteins other than G*α*_s_ in the endogenous context, particularly in extrapancreatic cell types.

### 2.3. *β*-Arrestin Coupling

Despite numerous studies characterizing *β*-arrestin interactions with GPCRs, only a limited number of reports investigate interactions between incretin receptors and arrestin proteins. Bioluminescence resonance energy transfer studies demonstrate both *β*-arrestin-1 and -2 interact with the GLP-1 receptor in an agonist dependent manner [97]. Classically, GPCR recruitment of GPCR kinases (GRKs) and *β*-arrestins is characterized as inducing desensitization of G-protein mediated signal transduction [98]; *β*-arrestin binding blocks G-protein mediated signaling and facilitates receptor internalization. However, emerging data suggest receptor activated *β*-arrestins can stimulate signaling pathways independently of G-protein activation [99]. Thus, *β*-arrestin signaling has physiologic consequences distinct from G-protein coupled signaling [99]. It is, therefore, of great interest to understand the functional outcome of *β*-arrestin regulation of the GLP-1 receptor.

In INS-1E insulinoma *β*-cells, siRNA knockdown of *β*-arrestin-1 reduces GLP-1 induced insulin secretion [30]. These experiments implicate *β*-arrestin-1 in GLP-1 receptor activity, but the mechanism responsible for the diminution of GLP-1 action is not explicitly characterized. An alternate explanation for lower GLP-1 stimulated insulin secretion could be reduced tonic inhibition from anti-insulinotropic G*α*_i_-coupled GPCRs resulting from *β*-arrestin-1 removal. Further, in this study, insulin secretion induced by glucose alone is severely attenuated by *β*-arrestin-1 knockdown making it difficult to establish direct causality between *β*-arrestin-1 knockdown and GLP-1 receptor dependent signaling.

In studies using MIN6 insulinoma *β*-cells, GLP-1 receptor stimulation is shown to induce a biphasic activation of ERK [100]. This effect is comprised of an initial cAMP-dependent transient activation of ERK and a prolonged *β*-arrestin-1 dependent activation of ERK [100]. *β*-arrestin-1 dependent ERK activity promotes Bad phosphorylation and consequently mediates prosurvival effects of GLP-1 receptor agonists on high glucose induced apoptosis. Because many biochemical mechanisms in MIN6 cells are operant in mouse islets, this report elegantly delineates separable pathways for GLP-1 receptor induced insulin secretion (G*α*_s_-cAMP axis) versus antiapoptotic signaling (*β*-arrestin-1 → p90RSK → Bad axis). It should be noted, though, that this study also contains experimental caveats; for example, the authors are unable to cause glucotoxic apoptosis in primary islet cultures and thus fail to validate the efficacy of GLP-1 anti-apoptotic signaling in their islet system.

*In vivo* analysis of insulin secretion in *β*-arrestin-1 knockout mice indicates glucose stimulated insulin secretion is reduced by over 80% [30, 100]. Accordingly, it is problematic to ascribe any physiologic alterations to GLP-1 receptor agonists in *β*-arrestin-1 knockout mice as being directly due to the GLP-1 receptor, given that *β*-arrestins are likely crucial signal regulatory proteins for hundreds of GPCRs. This is exemplified by the recent observation that *β*-arrestin-2 knockout mice are insulin resistant [101]. A key point to contextualize these studies is that although numerous reports in rodents show positive effects of GLP-1 or exendin-4 on pancreatic *β*-cell replication, *β*-cell mass, and function in preclinical models [102, 103], less data are available regarding GLP-1 agonist modulation of *β*-cell apoptosis or neogenesis in humans (discussed in [104]). One report does, however, demonstrate GLP-1 mediated attenuation of apoptosis and enhanced insulin responsiveness in an *ex vivo* human islet preparation [105]. Similarly, recent evidence demonstrates that GLP-1 receptor agonism induces *β*-cell replication in human islet grafts [106].

## 3. Structural Evaluation of the GLP-1 Receptor

Understanding the molecular mechanisms whereby peptide ligands induce GLP-1 receptor signaling should enhance new efforts to optimize small molecule activators of the GLP-1 receptor. Various approaches are being explored that may ultimately inform more rationale design strategies for small molecule GLP-1 receptor agonists. A comprehensive review of currently disclosed low molecular weight GLP-1 receptor activators is presented in this issue of *Experimental Diabetes Research*; see Willard et al. for review. Importantly, new strategies to exploit potential small molecule binding to the GLP-1 receptor are advancing as a result of progress in GPCR molecular and structural biology. Extensive site-directed mutagenesis studies have been carried out on the GLP-1 receptor as have structure activity studies on peptide GLP-1 ligands. It is beyond the scope of this review to cover these studies, however, we recognize that such efforts have been a valuable starting point in efforts to understand GLP-1 receptor biochemistry [107, 108].

### 3.1. Intramolecular Endogenous Peptide Agonists

Dong and colleagues proposed a novel activation mechanism for class B1 GPCRs [109] whereby peptide ligand interaction with the receptor induces a conformational change that exposes an intramolecular “endogenous agonist” epitope within the ECD of a GPCR. The “endogenous agonist” motif is proposed to act at the transmembrane domains to facilitate receptor activation. This hypothesis is largely derived from mutagenesis, peptide cross-linking, and molecular modeling studies using the secretin receptor [109]. In support of the concept, synthetic peptides derived from the ECD of the secretin receptor are shown to be low potency, high efficacy agonists of the secretin receptor. The minimized pharmacophore for the secretin receptor is a tri-peptide Trp^70^-Asp^71^-Asn^72^ [109]. Translation of this finding to the GLP-1 receptor identified an analogous peptide, Asn^63^-Arg^64^-Thr^65^-Phe^66^-Asp^67^ (NRTFD), as a low potency but fully efficacious GLP-1 receptor agonist [110]. Although these short peptides have low affinity and poor receptor selectivity, making them unlikely starting points for lead optimization and drug development, the molecules identify a potential binding site for compound action on the GLP-1 receptor. Structural elucidation of these features should be enlightening. The GLP-1 receptor “endogenous agonist” peptide maps to the *β*1 strand of the GLP-1 receptor ECD crystal structure [74]. However, analyses of these structural data suggest Asn^63^ is solvent exposed, but the majority of the peptide is not; these results make it difficult at this point to clearly understand the molecular mechanism proposed for the “endogenous agonist” peptides without further information around the structure or dynamics of the GLP-1 receptor. A subsequent report used an elegant cross-linking approach coupled with radiochromatography (see Section 3.3) to identify the site of action of the NRFTD peptide as being extracellular loop 3 in close proximity to transmembrane domain 6 [111]. The mechanism of action of the NRFTD peptide may be analogous to that of the “pepducins” [112]. Pepducins are short peptides derived from the intracellular loops of GPCRs that act allosterically to modulate receptor signaling [113].

### 3.2. Crystal Structure of the GLP-1 Receptor ECD

Receptor binding and functional studies show both GLP-1 (*K*_*d*_ = 0.3 nM) and exendin-4 (*K*_*d*_ = 0.1 nM) bind with high affinity and are full agonists at the GLP-1 receptor [26, 57]. In addition, competition binding studies suggest these peptides use the same ligand binding site within the receptor [114]. However, in experiments exploring the initial ligand-receptor binding event, data show exendin-4 binds the isolated soluble form of the GLP-1 receptor ECD with much greater affinity (13 nM) compared to GLP-1 (800 nM) [114, 115]. It was hypothesized that this phenomenon occurs because exendin-4 contains nine additional amino acids at its C-terminus that enable further binding contacts with the ECD [77, 115]. Importantly, the crystal structures of the ECD in complex with either bound GLP-1 or exendin-4_(9-39)_ show these peptides share a very similar mode of binding, and there is no interaction between the last seven residues of the nine amino acid C-terminal extension of exendin-4 with the ECD [74, 75] (Figure 3). Alternatively, biophysical studies suggest the higher propensity of exendin-4 to form helical conformations in solution compared to GLP-1 results in its enhanced binding affinity [73, 114]. However, this area is still in need of further exploration because other reports using a membrane-tethered form of the ECD demonstrate GLP-1 (IC_50_ = 160 nM) and exendin-4 (IC_50_ = 20 nM) bind the receptor with closer affinity [116]. Recent studies highlight this incongruity as exendin-4 (*K*_*d*_ = 0.9 *μ*M) and GLP-1 (*K*_*d*_ = 1 *μ*M) have equivalent affinities for the ECD, as measured by surface plasmon resonance [117]. While studies using purified forms of the ECD are informative, these latter results may highlight the relatively artificial nature of approaches using the soluble form of the ECD. Therefore, experimental methodologies allowing structural characterization of the full length, intact GLP-1 receptor are needed.

### 3.3. GLP-1 Receptor Photoaffinity Labeling

In the absence of a high resolution crystal structure of the full length GLP-1 receptor, photoaffinity labeling has been used as an alternate approach to identify potentially important ligand-receptor interactions. An inherent advantage of this technique is that whole cells expressing the native receptor or membrane preparations enriched with a receptor of interest are used so the receptor is folded and presented in its proper structural orientation [118–120]. These studies typically use a radio-labeled version of the natural ligand engineered to contain a photoreactive moiety such as *p*-benzoyl-l-phenylalanine (Bpa). Ultraviolet photolysis of a probe in complex with its receptor covalently labels residues of the receptor that are in close spatial approximations with important structural regions of the ligand. The labeled amino acids within the targeted receptor can then be identified using manual cycles of Edman degradation sequencing of isolated receptor fragments [121]. For the GLP-1 receptor, this technique has established spatial approximations between several ligand-receptor residues that likely occur in the “agonist occupied” receptor conformation. In studies intended to further assess the initial binding event, C-terminal residues, Ala^24^ and Gly^35^, of GLP-1 are shown to dock in close proximity near Glu^133^ and Glu^125^ of the ECD, respectively [122]. These data are in line with the 2.1 Å resolution crystal structure of the ECD-GLP-1 complex showing GLP-1 binding occurs via a continuous C-terminal *α*-helix formed by the sequence spanning Thr^13^ and Val^33^ with residues between Ala^24^ and Val^33^ directly interacting with the ECD [74]. Overall, these data are consistent with the initial binding event proposed by the “two-step” model. Somewhat surprisingly, results from studies testing GLP-1 probes with Bpa incorporated at positions 12 and 16 show docking of this region also near residues contained within the ECD. These data predict Phe^12^ and Val^16^ are positioned near Tyr^145^ and Leu^141^, respectively, of the ECD, sites located in the distal region of the ECD immediately upstream of the first transmembrane segment of the receptor [123, 124].

Importantly, photoaffinity cross-linking studies have also established potential contacts between the extracellular loops of the receptor with residues of GLP-1. This work is helping provide better insights into the orientation of the ligand bound N-terminal region of GLP-1 with the receptor core. For studies aimed at identifying structural elements involved with the “second step” of GLP-1 binding and receptor activation, an N-terminus labeled photo-labile GLP-1 probe was generated. Because changes to the N-terminal His^7^ are not well tolerated [125], a probe with Bpa N-terminally attached to His^7^ (at a new position 6) was used to better understand spatial approximations between the most N-terminal residues of GLP-1 and the receptor. These data show the N-terminus positions near Tyr^205^ in the first extracellular loop of the receptor [123]. Similarly, using a mid-region position 20 probe, Trp^297^ within the second extracellular loop of the receptor positions within close proximity to Leu^20^ of GLP-1 [124]. Taken together, X-ray structural data of the ECD in complex with the C-terminus of GLP-1 and the cumulative results from photoaffinity labeling studies provide experimentally derived information with which to generate a more accurate molecular model of the ligand binding pocket for the GLP-1 receptor [124].

### 3.4. Emerging Biochemical Technologies

Although progress is continuing, integrated strategies pairing classic receptor pharmacology with newer biophysical and structural biology techniques are needed to progressively refine a model for GLP-1 receptor activation. New techniques aimed at understanding ligand dependent conformational changes in the GLP-1 receptor should aide small molecule discovery pursuits. One emerging approach is the application of solution phase peptide amide hydrogen/deuterium exchange (HDX) coupled with mass spectrometry (MS) for the study of GPCRs. In contrast to photoaffinity labeling, HDX-MS does not require generating probes that require incorporation of a bulky moiety, such as the hydrophobic Bpa, and must retain the binding and activation properties of the natural ligand. Alternatively, HDX-MS is based on the principle that for proteins in solution, amide bond hydrogen atoms are exchangeable, and differences in the rate of exchange are indicative of local accessibility and thus can reflect the conformational status of a protein [126, 127]. Deuterium incorporation into the peptide backbone increases protein mass, and upon protease cleavage, the location and degree of hydrogen/deuterium exchange can be mapped via MS analysis. Although technically difficult to apply to membrane proteins, methods have advanced that better enable purification procedures for isolating GPCRs using detergents that help maintain native structure, protein solubility, and functional activity. From these advances, HDX-MS has now been used to study ligand induced conformational changes of the *β*2 adrenergic receptor [128, 129]. These biochemical studies demonstrate that distinct receptor conformations are elicited by ligands with different intrinsic efficacies. Further, an elegant parallel MS approach has utilized covalent derivatization of cysteine and lysine residues with stable isotope labeled reactive functionalities to assess dynamic conformational changes elicited by *β*2 adrenergic receptor ligands [130]. These studies highlight the diversity of receptor conformations induced by ligands with apparently similar functional capacities. Together, this work provides further experimental evidence for the existence of multiple ligand specific conformations of GPCRs.

### 3.5. Recent Advances in GPCR Crystallography

The first GPCR crystal structure determined was that of Rhodopsin in 2000 [131]. While informative about the general principles of GPCR structure, and having utility as a homology model template for closely related class A GPCRs, this information has not significantly impacted drug discovery activities for class B1 GPCRs. However, recent advances in GPCR biochemistry and macromolecular crystallography have accelerated the pace of structure determination for this important target class [132]. Since 2007, multiple new class A GPCR structures have been determined, including adrenergic, adenosine, chemokine, dopamine, and histamine receptors [132]. In many cases, structures of multiple ligands with cognate GPCRs are solved. Moreover, the recent determination of a G-protein bound complex of an activated GPCR represents a landmark achievement of GPCR crystallography [133]. Several new technological advances have been developed to facilitate these pursuits, including novel detergents [134], creative receptor-fusion proteins [135], camelid nanobodies [136], and lipidic cubic phase crystallization [137]. Consequently, we anticipate that significant effort may now turn toward determining the crystal structures of class B1 GPCRs.

## 4. Conclusions

It is clear the GLP-1/GLP-1 receptor axis is a key physiologic regulator of glucose metabolism, and diabetic patients treated with degradation resistant GLP-1 receptor peptide agonists, exenatide and liraglutide, experience improved glucose homeostasis. Therefore, efforts to identify orally active small molecule GLP-1 receptor agonists are justifiably being pursued. While several scaffolds are now reported (see Willard et al. in this issue of *Experimental Diabetes Research*), high-throughput screening campaigns and other discovery approaches have largely failed to identify quality chemical starting points that have been successfully optimized into therapeutic agents. The lack of apparent success is likely due to the inherent difficulty of lower molecular weight, nonpeptide molecules to mimic the complex nature of peptide ligand binding to the GLP-1 receptor ECD and transmembrane regions needed to induce intrinsic structural changes in the receptor to elicit signal transduction.

Fortunately, the availability of peptide ligands for the GLP-1 receptor enables very detailed assessment of GLP-1 receptor signaling pathways, and newer biophysical techniques are helping interrogate the integral mechanisms involved in receptor activation. Further, advances in structural biology methodologies for GPCRs are now rapidly occurring, and application of these techniques to class B1 GPCRs will be groundbreaking. Assimilation of structural information for the full length, intact GLP-1 receptor and improved assay systems with which to monitor the GLP-1 receptor in different conformational states will likely be critical to advancing nascent efforts to identify GLP-1 receptor small molecule ligands. Once novel compounds emerge, it will be important to optimize molecules using testing schemes that incorporate signal transduction mechanisms of GLP-1 physiology, especially GLP-1 receptor stimulation of cAMP production to enhance glucose dependent insulin secretion.
